# InRegPep: a web server for modeling of parallel beta-sheet fibrillar aggregates of amyloidogenic peptides

**DOI:** 10.3389/fmolb.2026.1908797

**Published:** 2026-10-16

**Authors:** Wojciech Puławski, Szymon Niewieczerzał, Andrzej Koliński, Grzegorz Firlik, Michał Koliński

**Affiliations:** 1 Bioinformatics Laboratory, Mossakowski Medical Research Institute, Polish Academy of Sciences, Warsaw, Poland; 2 Biological and Chemical Research Centre, University of Warsaw, Warsaw, Poland; 3 Faculty of Chemistry, University of Warsaw, Warsaw, Poland

**Keywords:** amyloid polymorphism, multiscale modeling, peptide aggregation, peptide-peptide docking, protofilament modeling

## Abstract

Predicting the structures of peptide or protein aggregates that assemble into amyloid protofilaments remains one of the most challenging tasks in computational structural biology. Computational difficulties arise mainly from the large size of the modeled systems and the extensive timescales of aggregation processes. The InRegPep web server is designed for the multiscale structural modeling of peptide aggregates that preferentially adopt in-register parallel β-sheet architectures. The method employs the coarse-grained CABS model, which enables the assembly of a large number of aggregates composed of five interacting peptides. The best models are selected using clustering techniques combined with energy-based and model-symmetry criteria. Top-scoring models are subsequently refined at the all-atom level and further assessed using molecular dynamics simulations. Finally, short protofilament structures are assembled for conformationally stable aggregates. The performance of the InRegPep server was evaluated using a benchmark dataset comprising 15 experimentally determined protofilament structures. For 10 of the 15 systems, the best-performing model among the 10 top-scoring predictions achieved an RMSD below 5 Å. The resulting models provide a valuable foundation for subsequent studies, including investigations of conformational polymorphism, mutational design, and downstream experimental or computational validation.

## 1 Introduction

Under certain conditions, proteins and peptides can misfold and aggregate into insoluble supramolecular assemblies known as amyloid fibrils (Rambaran and Serpell, 2008). The formation of these structures is associated with a wide range of physiological and pathological processes and is linked to numerous neurodegenerative and systemic disorders, including Alzheimer’s disease, Parkinson’s disease, Creutzfeldt–Jakob disease, and type II diabetes (Ross and Poirier, 2004). Fibrillar structures consist of one or more laterally associated protofilaments (Taylor and Staniforth, 2022), which are typically composed of stacked peptide or protein layers arranged in specific architectures, most commonly in-register parallel β-sheets (Eisenberg and Sawaya, 2017). However, alternative arrangements, such as antiparallel or out-of-register configurations, as well as other more complex structural aggregates, have also been reported (Makin and Serpell, 2005). The structural organization of amyloid aggregates strongly affects their biological properties, such as toxicity, ability to seed new aggregates, and capacity to propagate (Taylor and Staniforth, 2022; Tycko, 2015). Understanding the structural properties of amyloid assemblies is therefore crucial for gaining insight into the aggregation process and for the development of therapeutic strategies. It may guide the design of aggregation inhibitors, modulators of fibril growth, and molecules capable of selective binding to specific fibril forms. Additionally**,** the rational engineering of peptides with altered aggregation propensity can be exploited in both biomedical and nanotechnological applications.

In recent years, experimental studies of amyloid structures have undergone substantial advancements, driven primarily by developments in cryo-electron microscopy (cryo-EM), atomic force microscopy (AFM), and solid-state nuclear magnetic resonance (ssNMR) spectroscopy. These techniques have enabled the determination of an increasing number of high-resolution fibrillar structures and have established conformational polymorphism as a pervasive feature of amyloid assemblies, whereby a single peptide sequence can adopt multiple structurally distinct fibril architectures depending on environmental and experimental conditions. Concurrently, dedicated databases such as Amyloid Explorer, Amyloid Atlas, and others (Iglesias et al., 2024; Burdukiewicz et al., 2023) have emerged, facilitating systematic structural comparison and supporting experimental and computational analyses of these systems. Despite these advances, the current repertoire of high-resolution amyloid structures remains limited in contrast to the extensive diversity of amyloidogenic sequences and the wide range of physicochemical conditions under which fibrillation occurs.

Theoretical approaches have become an essential complement to the experimental efforts outlined above. A wide range of strategies has been explored, spanning physics-based simulations and machine learning methods. The AlphaFold method has revolutionized the modeling of individual folded proteins and protein complexes (Jumper et al., 2021; Abramson et al., 2024); however, predicting the structures of large, heterogeneous, and polymorphic aggregates such as amyloid fibrils remains a major challenge (Pinheiro et al., 2021; Guo et al., 2025). While machine learning methods perform impressively in protein structure prediction (Jumper et al., 2021; Abramson et al., 2024), they often struggle to capture large-scale assembly processes, structures of multi-subunit assemblies presenting alternative conformational states, and long-timescale dynamics (Szczepski and Jaremko, 2025). Amyloids formed by the aggregation of relatively short and unstructured peptides are also challenging for machine learning methods (McDonald et al., 2023). Recent benchmarking of AlphaFold2 on a large set of 588 short peptide structures indicated that the method performs best for peptides with well-defined secondary structure, but shows reduced accuracy in predicting conformations for peptides with extended coil or flexible regions (McDonald et al., 2023).

Artificial intelligence–based methods typically aim to identify a single native conformation corresponding to the global minimum of the energy landscape; although, very recently, AI studies of polymorph landscapes of amyloid fibrils were also reported using RibbonFold (Guo et al., 2025). This method builds on AlphaFold2 by incorporating parallel in-register constraints into its template module, along with a polymorphism loss function designed to capture structural diversity among folds.

Other computational strategies have also been proposed for studying amyloids. For example, a hybrid method combining threading techniques with machine learning has been developed to predict peptide amyloidogenicity (Wojciechowski and Kotulska, 2020). Tools specifically designed for the computational prediction of amyloid fibril structures, such as FibPredictor (Tabatabaei Ghomi et al., 2016) and CreateFibril (Smaoui et al., 2013), have also been introduced. In addition, a wide range of simulation-based strategies has been applied to investigate the aggregation process (Strodel, 2021; Dey and Biswas, 2023) and to determine fibril structures (Sharma and Dill, 2021).

Computational modeling, particularly coarse-grained (CG) simulations, provides a valuable framework for sampling a diverse ensemble of conformational states (Chen et al., 2018; Kmiecik et al., 2016; Puławski et al., 2023; Puławski et al., 2025). These methods enable a more explicit treatment of intermolecular interactions and facilitate exploration of extensive conformational spaces, albeit often at the expense of atomic-level resolution or increased computational cost. Hybrid and multiscale strategies that combine efficient sampling with physically grounded scoring functions appear especially promising for modeling amyloid assemblies, including their distinct polymorphic conformations (Chen et al., 2018; Puławski et al., 2023; Puławski et al., 2025; Puławski and Dzwolak, 2022).

In this work, we present InRegPep, a computational workflow designed to model peptide aggregates that preferentially adopt in-register parallel β-sheet architectures. Rather than attempting to predict a single “correct” structure, our method generates ensembles of candidate models. This makes it possible to capture structural variability and to identify the most stable and representative conformations. In this multiscale approach, InRegPep integrates CG modeling based on the CABS framework (Kmiecik et al., 2016; Kolinski, 2004; Kurcinski et al., 2020) with clustering, symmetry evaluation, reconstruction to all-atom resolution and subsequent molecular dynamics refinement. The proposed protocol provides a systematic and scalable framework for constructing protofilament models for putatively amyloidogenic peptide sequences (Puławski et al., 2023; Puławski et al., 2025; Koliński et al., 2021). This ensemble-based strategy offers a solid foundation for studying amyloid polymorphism, guiding mutational experiments, and supporting further experimental validation.

## 2 Materials and methods

### 2.1 Server description

Main stages of the multiscale modeling pipeline implemented in the InRegPep server (https://bioinfo.imdik.pan.pl/inregpep/) include: (1) Input: Input data include the amino acid sequence of the amyloidogenic peptide and the choice of simulation parameters. Sequences ranging from 10 to 100 amino acids can be submitted. Simulation parameters include the secondary structure assignment, the number of docking runs, the number of Monte Carlo cycles, the temperature range, the pH value, and an optional setting for adding restraints that mimic intrachain disulfide bridges. (2) Restraints: Distance restraints are imposed between side-chain united atoms of corresponding residues in neighboring peptide chains to favor an in-register, parallel peptide arrangement. Optionally, additional restraints are generated to mimic intrachain disulfide bonds, as specified by the user. (3) Peptide docking: Five identical peptides are docked simultaneously to form aggregates. The docking simulation is performed using the CABS CG framework (Kurcinski et al., 2020; Kurcinski and Kolinski, 2007), which enables fast and efficient sampling of conformational space and generates a large ensemble of plausible aggregate structures represented by Cα-traces. All peptides remain fully flexible throughout the docking process. Up to 100 independent docking runs can be performed for each system, generating up to 1,000,000 unique aggregate models. (4) Model scoring: Twenty unique models, each composed of five identical peptides, are selected from the generated set of models. The selection is based on a combination of energy-based criteria, structural symmetry [quantified by the pcaRMSD parameter (Puławski et al., 2023)], and a k-means clustering procedure. (5) Refinement: The selected peptide aggregates are reconstructed into all-atom representations. The atomistic models undergo geometry optimization, including energy minimization followed by molecular dynamics simulation (MD) using OpenMM (Eastman et al., 2023). The top 10 highest-scoring models are selected using structural parameters derived from MD trajectories. Conformational stability is evaluated based on observed changes of the pcaRMSD and RMSD values, intermolecular contact analysis, and secondary structure conservation during the MD simulation. (6) Output: After the calculations are completed, analyses are performed based on MD trajectories. The output includes results for the 10 highest-ranked structures. Models are evaluated in terms of conformational stability and the translational symmetry of interacting peptides. The server provides plots of RMSD, per-residue RMSF, pcaRMSD (including CABS energy vs. pcaRMSD across docking trajectories), β-sheet content, and interlayer contact data. Each aggregate model is classified as “plausible” or “unstable.” For plausible models, protofilament structures are proposed. Protofilament models consisting of 15 peptides are assembled based on structural regularities observed in predicted aggregates. The peptide from the central layer is used as the building block for protofilament assembly. Successive layers are generated along the protofilament’s long (Z) axis by replicating this peptide and positioning each copy using a transformation matrix derived from the aggregate structures. The resulting protofilament model is then subjected to a geometry optimization procedure. The InRegPep modeling pipeline is illustrated below in Figure 1.

**FIGURE 1:**
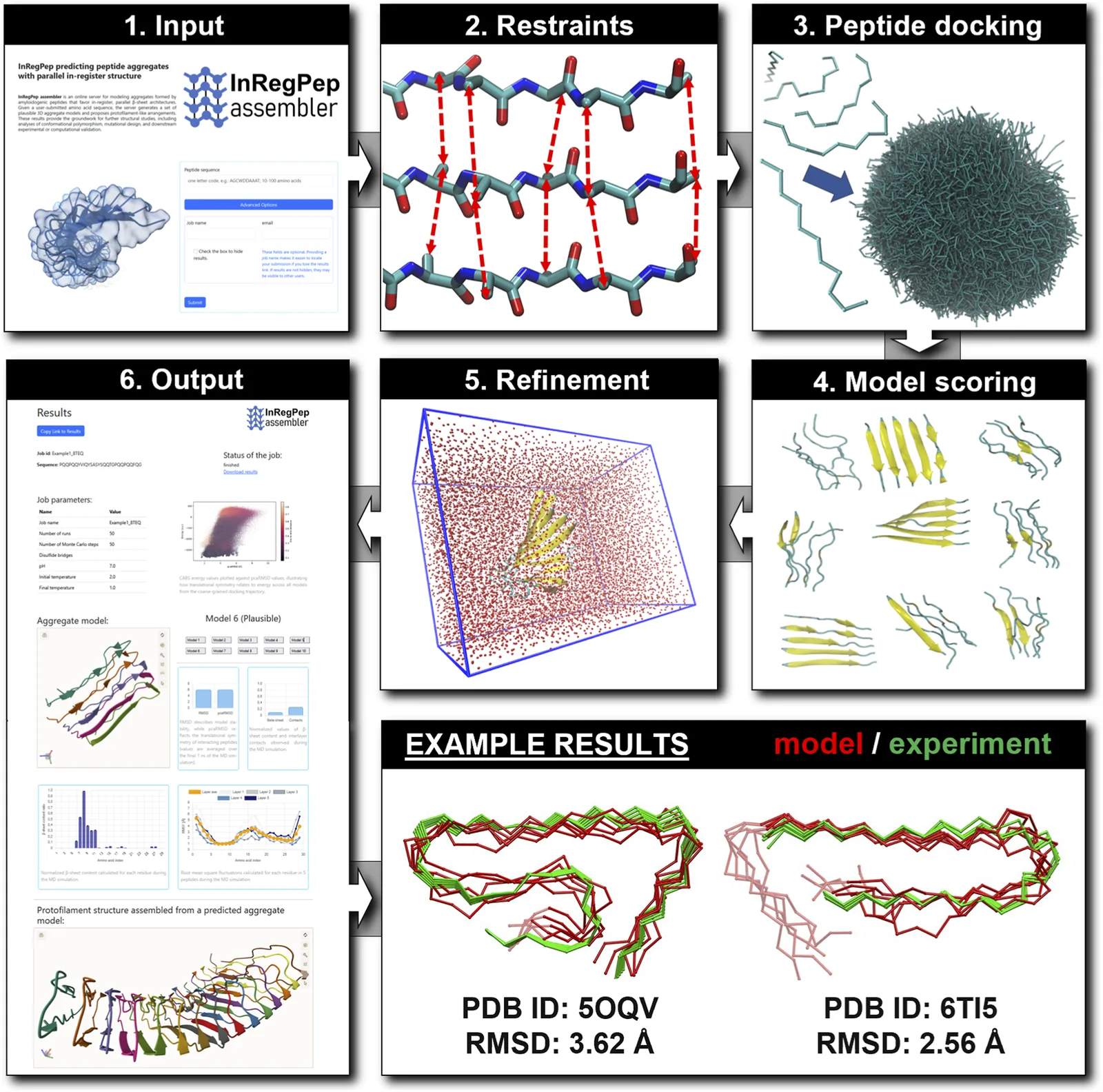
Main stages of the InRegPep multiscale modeling workflow. Panels 1–6 illustrate the main stages of the modeling pipeline. The bottom-right panel presents models generated for two polymorphic conformations of Aβ42/Aβ40 (PDB ID: 5OQV (Schmidt et al., 2015), and PDB ID: 6TI5 (Cerofolini et al., 2020). Red trace indicates the best model found in the trajectory, whereas green denotes the experimental structure. For the first model, the RMSD value is calculated for the entire peptide, while for the second, the RMSD is calculated only for the fragment corresponding to the experimental reference structure.

### 2.2 The CABS coarse-grained multichain model

The CABS is a well-established model for studies of protein structure and dynamics (Kmiecik et al., 2016; Kolinski, 2004). It has been successfully applied to peptide–peptide (Puławski et al., 2023; Puławski et al., 2025; Koliński et al., 2021) and peptide–protein (Kurcinski et al., 2020; Badaczewska-Dawid et al., 2021; Koliński et al., 2020) docking (Kurcinski et al., 2020; Badaczewska-Dawid et al., 2021; Koliński et al., 2020), protein folding and structure prediction (Kmiecik and Kolinski, 2007), including modeling of membrane proteins (Pulawski et al., 2016; Kmiecik et al., 2014; Kolinski and Filipek, 2010), and studies of protein dynamics (Kmiecik et al., 2018; Jamroz et al., 2014). The CABS method, and its extension for simulation of multichain systems (Kurcinski et al., 2020; Kurcinski and Kolinski, 2007; Kurcinski et al., 2019) use a specific CG representation of protein structures that enables efficient simulation of protein complexes, including peptide aggregates (Kmiecik et al., 2016; Puławski et al., 2023; Puławski et al., 2025; Kolinski, 2004; Koliński et al., 2021). In this model, each residue of the polypeptide chain is represented by up to four pseudo-atoms: Cα, Cβ, a united atom placed at the center of the remaining portion of the side chain, and a pseudo-atom used for the definition of an ersatz main-chain hydrogen bond. A key feature of the model is the placement of Cα atoms on a dense three-dimensional lattice, while the remaining pseudo-atoms are positioned off-lattice based on the geometry of the backbone. This hybrid representation preserves near-atomistic resolution of the protein backbone (∼0.5 Å) while significantly reducing computational complexity. The lattice-based discretization of the backbone plays a crucial role in enhancing sampling efficiency. Because Cα positions are restricted to lattice nodes, conformational space can be explored through a large number of predefined local moves that are computationally inexpensive. At the same time, the lattice is sufficiently dense to avoid directional biases, ensuring realistic geometries of secondary structure elements such as β-strands and helices, regardless of their mutual orientations. This feature is particularly important for modeling amyloid aggregates, where precise backbone alignment and hydrogen-bonding patterns define in-register, parallel β-sheet architectures.

CABS force field (Kolinski, 2004) is based on knowledge-based statistical potentials that encode general principles of protein structural regularities, including excluded volume, side-chain interactions, and directional hydrogen bonding. This unique interaction model includes terms that favor not only helical but also extended β-strand conformations and inter-chain contacts, which are essential for stabilizing amyloid-like arrangements. In multimer simulations, these interactions naturally facilitate the formation of ordered β-sheet assemblies while still allowing for structural variability.

Conformational sampling in the CABS model is driven by a Monte Carlo scheme employing local moves and the Metropolis acceptance criterion. This approach enables efficient exploration of the conformational landscape over long effective timescales. The combination of lattice-based representation and stochastic sampling allows the system to overcome energy barriers and explore multiple alternative conformational states. As a result, the model is particularly well-suited for studying amyloid systems, which are inherently polymorphic and can adopt multiple competing structural arrangements. Importantly, the CABS multimer model enables the simultaneous simulation of multiple interacting peptide chains, allowing the direct modeling of aggregate formation and their structural organization (Puławski et al., 2025; Kurcinski et al., 2020; Koliński et al., 2021; Kurcinski and Kolinski, 2007; Kurcinski et al., 2019; Ciemny et al., 2017). The efficient sampling of diverse conformational states makes it especially suitable for identifying in-register, parallel β-sheet architectures, while also capturing alternative packing modes that may correspond to different polymorphic forms.

## 3 Results and discussion

### 3.1 Benchmarking

The performance of the InRegPep server was evaluated using a benchmark dataset composed of 15 experimentally determined protofilament structures obtained by nuclear magnetic resonance (NMR) spectroscopy and cryo-electron microscopy (cryo-EM). All selected structures exhibited an in-register, parallel β-sheet architecture. The peptide lengths ranged from 22 to 40 amino acid residues. All simulations were performed using 100 docking runs and 100 Monte Carlo cycles, while the remaining parameters were set to their default values on the server input page.

The predicted aggregates were constructed from five simultaneously docked peptide chains. The resulting models were compared with their corresponding experimental structures. Structural similarity was quantified using root-mean-square deviation (RMSD), calculated after superimposition of the predicted and experimental protofilament structures. The results are summarized in Table 1. Columns 3–8 report: (i) the lowest RMSD observed across all models provided by the server, (ii) the lowest RMSD among the top 10 best-scoring models, (iii) the RMSD of the single best-scoring model (iv–vi) the mean, median, and standard deviation of RMSD across all generated models, respectively. For 10 out of 15 systems, the best model within the set of 10 top-scoring models achieved an RMSD below 5 Å, indicating that in approximately two-thirds of the analyzed cases, the method produced accurate protofilament models. The correlation between pcaRMSD and RMSD values, as well as the distribution of RMSD values calculated for all models generated during the benchmark, is presented in Supplementary Figure S1. Additional data showing the number of high-quality models obtained for each benchmark system are provided in Supplementary Table S1. Structural characteristics of the benchmark models, including β-sheet content and twist angle, are reported in Supplementary Table S2.

**TABLE 1:** Prediction results for a benchmark comprising 15 experimental protofilament structures.

N.O.	PDB ID	RMSD [Å]						Peptide length	Peptide sequence
		Best from all	Best from top10	Best scored	Mean	Median	Standard deviation		
1	8TEQ (Rosenberg et al., 2023)	2.81	2.84	5.00	10.42	7.92	5.52	29	PQQPQQYVIQYSASYSQQTGPQQPQQFQG
2	2NNT (Ferguson et al., 2006)	2.74	2.97	4.59	10.48	7.94	6.49	31	MGATAVSEWTEYKTADGKTFYYNNRTLESTW
3	2E8D (Iwata et al., 2006)	2.52	3.36	5.12	7.59	5.71	3.94	22	SNFLNCYVSGFHPSDIEVDLLK
4	6TI5 (Cerofolini et al., 2020)	2.40	3.64	5.17	9.62	6.51	5.78	30	EVHHQKLVFFAEDVGSNKGAIIGLMVGGVV
5	6VPS (Hervas et al., 2020)	3.01	3.71	3.77	11.64	9.13	6.84	31	QLHQQQHQQQHQQHQQHQQQQQLHQHQQQLS
6	2MPZ (Sgourakis et al., 2015)	2.50	4.09	4.96	8.61	6.05	4.89	26	QKLVFFAENVGSNKGAIIGLMVGGVV
7	8EZD (Lee et al., 2023)	3.55	4.44	18.31	10.25	7.88	4.85	31	VHHQKLVFFAEDVGSNKGAIIGLMVGGVVIA
8	6ZRQ (Gallardo et al., 2020)	3.67	4.62	4.62	9.78	8.19	3.58	23	FLVHSGNNFGAILSSTNVGSNTY
9	6N37 (Cao et al., 2019)	3.73	4.84	17.9	14.47	13.88	5.86	36	NFGAFSINPAMMAAAQAALQSSWGMMGMLASQQNQS
10	8AZ1 (Wilkinson et al., 2023)	3.34	4.91	8.67	10.57	9.33	3.54	25	ANFLVHSGNNFGAILSSTNVGSNTY
11	8SEK (Fernandez et al., 2024)	5.04	5.41	9.22	12.04	9.41	5.03	32	GYEVHHQKLVFFAEDVGSNKGAIIGLMVGGVV
12	6UUR (Glynn et al., 2020)	3.03	5.47	17.01	16.20	16.96	7.11	40	KTNMKHMAGAAAAGAVVGGLGGYMLGSAMSRPIIHFGSDY
13	2LMN (Paravastu et al., 2008)	4.12	5.89	7.75	10.90	8.19	5.06	32	GYEVHHQKLVFFAEDVGSNKGAIIGLMVGGVV
14	8OVM (Frieg et al., 2024)	4.40	7.30	16.15	12.32	12.37	3.61	36	RHDSGYEVHHQKLVFFAEDVGSNKGAIIGLMVGGVV
15	2BEG (Lührs et al., 2005)	3.29	7.38	9.00	10.23	8.36	3.73	26	LVFFAEDVGSNKGAIIGLMVGGVVIA

Analysis of the docking trajectories shows that aggregate structures closely resembling experimental conformations are included in the predicted model ensemble for almost all systems (except 8SEK). This suggests that the sampling procedure is generally sufficient to explore the relevant regions of the conformational landscape for all analyzed aggregates. However, identifying near-native structures among a large set of generated models remains a difficult task (Zalewski et al., 2021). In most cases, models with low RMSD values are not found among the top-scoring solutions, suggesting limitations in the scoring procedure. The scoring problem is widely recognized as one of the most challenging aspects of protein structure prediction (Kmiecik et al., 2016; Zalewski et al., 2021), particularly for systems such as amyloid aggregates, which exhibit subtle energetic differences between alternative conformations and a high degree of structural polymorphism. Nevertheless, near-native structures are included among the top 10 scored solutions for most of the analyzed systems.

The accuracy of the modeling pipeline implemented in the InRegPep server was further validated through test predictions of additional protofilament structures for which experimental data are available, as reported in previous studies (Puławski et al., 2023; Koliński et al., 2021). A similar computational protocol was also successfully used to accurately predict the structure of the insulin protofilament (Puławski et al., 2025).

It is important to note that the validation of the method was conducted on systems composed of relatively short peptides, and even though the upper limit of the peptide length that can be subjected to prediction is 100 residues, the method performs better for aggregates composed of shorter peptides. We also tested the method on longer peptides for which experimental structures are known, including different polymorphs (i.e., the tau protein, 88 amino acids in length); however, the scoring function was not able to deliver a set of the 10 best-scored models that included a conformation close to the reference experimental structures. Nevertheless, structures closely resembling the different polymorphs were found in the generated trajectory, which is further discussed in the next section.

### 3.2 Exploring amyloid polymorphism using the InRegPep server

Rapidly developing artificial intelligence-based methods such as AlphaFold, while highly successful in predicting the structures of folded globular proteins and their complexes, still face significant limitations when applied to amyloid aggregates (Pinheiro et al., 2021; Guo et al., 2025) and multichain peptide assemblies (McDonald et al., 2023). These methods are typically trained on datasets dominated by well-folded, monomeric proteins and are therefore focused on predicting a single most probable structure corresponding to a global energy minimum. Consequently, capturing structural heterogeneity, conformational multiplicity, and weakly defined energy landscapes—hallmark features of amyloid aggregation—remains challenging. Moreover, amyloid fibrils are formed by repetitive stacking of flexible or partially disordered peptide chains, often involving subtle differences in inter-chain packing registry. Such features are difficult for AI models to resolve accurately, particularly in the absence of strong evolutionary signals or multiple sequence alignments, which are key inputs for methods such as AlphaFold. Short peptides and intrinsically disordered regions—common in amyloid systems—are especially challenging for AI-based approaches, as demonstrated in benchmarking studies showing limited prediction accuracy for such sequences (McDonald et al., 2023). It should also be noted that current AI-based approaches do not explicitly model the process of aggregation or the cooperative interactions between multiple chains over extended timescales.

The CABS model explicitly accounts for intermolecular interactions and enables direct simulation of multichain assemblies. Its efficient sampling strategy allows exploration of a wide range of alternative conformations rather than converging to a single solution. This makes it particularly well-suited for identifying in-register, parallel β-sheet architectures, while also capturing alternative packing modes corresponding to different polymorphic forms. The ability to generate and analyze ensembles of structures provides a more realistic description of amyloid systems and their conformational diversity. Knowledge of probable association pathways, including structural representations of assembly intermediates, may be useful for certain aspects of molecular biology and medicine. In summary, the balance between computational efficiency, physically grounded interactions, and extensive conformational sampling makes the CABS framework a powerful tool for exploring the structural landscape of amyloid aggregates, complementary to current AI-based prediction approaches.

The structural basis of polymorphism originates from the complex and relatively rugged energy landscape associated with amyloid assembly. Unlike globular proteins, which are generally optimized to fold into a unique native structure, amyloid-forming peptides may populate multiple competing minima corresponding to alternative fibrillar assemblies that can differ in morphology, stability, and biological properties (Tycko, 2015; Adamcik and Mezzenga, 2018; Paravastu et al., 2008). Recent theoretical studies have emphasized that amyloid structures should be viewed as ensembles of accessible conformational states rather than as single well-defined architectures. In particular, the recently developed RibbonFold approach demonstrated that even highly ordered in-register parallel β-sheet assemblies may populate a limited but significant set of alternative polymorphic forms (Guo et al., 2025). Previous computational studies of Aβ fibrils showed that several distinct protofilament topologies can remain structurally stable during molecular dynamics simulations despite exhibiting different patterns of intermolecular packing and side-chain interactions. Such observations indicate that multiple structurally distinct assemblies may coexist within similar energetic ranges, further highlighting the necessity of computational methods capable of sampling diverse fibrillar conformations (Buche et al., 2005). These findings further support the concept that amyloid fibrils occupy polymorphic structural landscapes whose exploration is essential for understanding aggregation mechanisms and disease-related structural diversity.

The InRegPep server provides a practical framework for investigating amyloid polymorphism within the class of in-register parallel β-sheet assemblies. Rather than converging toward a single structural solution, the protocol generates extensive ensembles of aggregate models and evaluates them using energy-based criteria, structural symmetry measures, clustering procedures, and molecular dynamics refinement. This ensemble-oriented strategy naturally enables the identification of multiple plausible aggregate architectures for a given peptide sequence. The ability to capture alternative packing modes and distinct in-register parallel β-sheet organizations is particularly valuable because such structural variability represents the molecular foundation of amyloid polymorphism.

The polymorphic ensembles generated by InRegPep can therefore be viewed as approximations of the underlying amyloid conformational landscape. Analysis of these ensembles allows the identification of dominant structural motifs, alternative protofilament arrangements, and potentially competing aggregation pathways. Consequently, InRegPep extends beyond conventional structure prediction and serves as a useful platform for studying the structural diversity of amyloid assemblies. The InRegPep server typically generates 10 independent aggregate models, each representing a plausible alternative conformation. Analysis of docking trajectories for systems such as tau protein and Αβ42 shows that the generated ensemble includes structures closely resembling experimentally determined polymorphic conformations or, at a minimum, captures key structural motifs observed in fragments of these structures (see Figures 2, 3).

**FIGURE 2:**
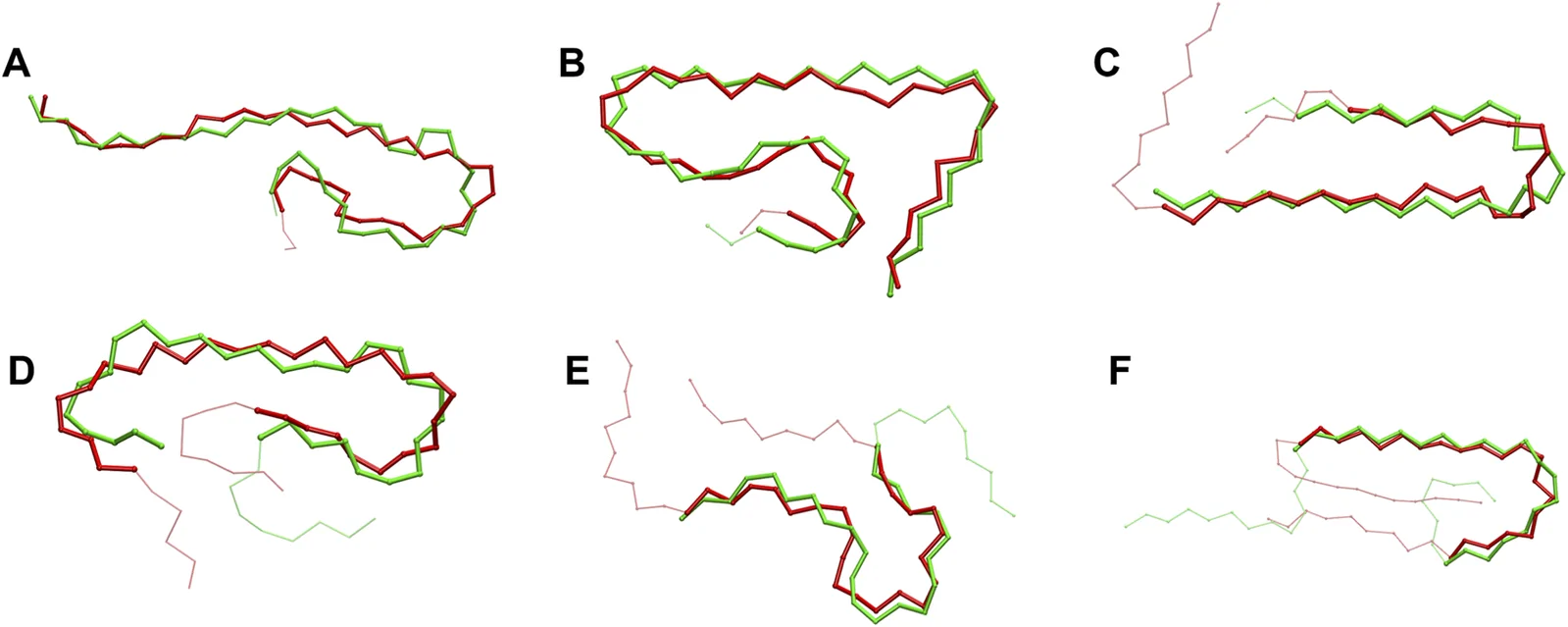
Comparison of experimental Aβ42/Aβ40 polymorphic structures (shown in green) with conformations generated by docking simulations (shown in red). For clarity, only single chains from fibrillar complexes are presented using Cα trace representations. RMSD values were calculated using 5, 4, or 3 chains, depending on the reference structure. Predicted models contain 42 residues, whereas reference structures are shown at their deposited lengths in the PDB. Thicker lines indicate fragments included in RMSD calculations. The panels show: **(A)** PDB ID: 2M4J (Lu et al., 2013), RMSD = 3.44 Å (residues 1–39); **(B)** PDB ID: 5OQV (Schmidt et al., 2015), RMSD = 3.48 Å (residues 1–40); **(C)** PDB ID: 6TI5 (Cerofolini et al., 2020), RMSD = 1.98 Å (residues 11–26); **(D)** PDB ID: 7F29 (Liu et al., 2021), RMSD = 3.04 Å (residues 6–34); **(E)** PDB ID: 2MXU (Xiao et al., 2015), RMSD = 1.91 Å (residues 11–33); and **(F)** PDB ID: 8OWD (Frieg et al., 2024), RMSD = 1.54 Å (residues 13–33). In general, the predicted conformations reproduce substantial portions of the corresponding reference structures with good accuracy. The main inaccuracies in the models are observed in the N- and C-terminal regions. In particular, in the case of 8OWD (a lipid-associated Aβ40 fibril), structural similarity is limited to a relatively short segment (∼20 residues). Note that the RMSD values reported here differ from those in the benchmark analysis for corresponding systems, as they were calculated only for the peptide fragment showing the greatest structural similarity to the reference structure, rather than over the full length of the simulated peptide.

**FIGURE 3:**
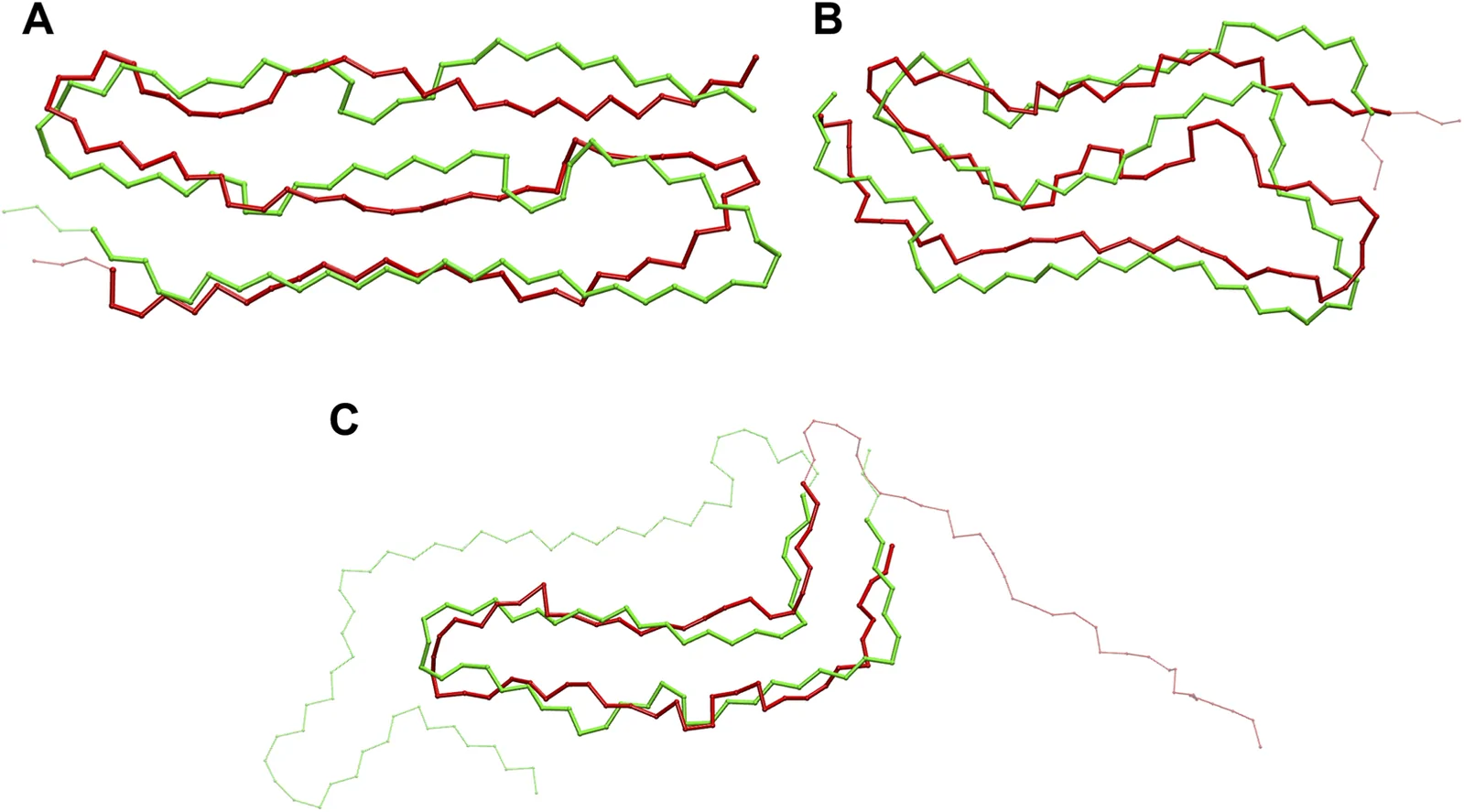
Comparison of tau protein polymorphic structures (shown in green) with conformations generated by docking simulations (shown in red). For clarity, only single chains from fibrillar complexes are presented using Cα trace representations. RMSD values were calculated using 5, 4, or 3 chains, depending on the reference structure. Predicted models contain 88 residues. Whereas reference structures are shown at their deposited lengths in the PDB. Thicker lines indicate fragments included in RMSD calculations. The panels show: **(A)** PDB ID: 8TTN (Mammeri et al., 2024), RMSD = 5.00 Å (residues 275–360); **(B)** PDB ID: 7P66 (Shi et al., 2021), RMSD = 6.18 Å (residues 279–367); and **(C)** PDB ID: 7U0Z (Chang et al., 2022), RMSD = 6.17 Å (residues 275–330).

After successful job completion, the InRegPep server provides a downloadable zipped file archive containing the resulting data. This archive includes a model ensemble in Cα-trace representation, with distinct aggregate conformations corresponding to the centroids of clusters identified from independent docking trajectories. For this purpose, k-means clustering is performed on low-energy models that exhibit high translational symmetry of interacting peptides within the predicted aggregate (see Supplementary Material, section: Usage).

## 4 Conclusion

InRegPep is a dedicated web-based platform for modeling aggregates formed by amyloidogenic peptides that preferentially adopt in-register, parallel β-sheet architectures. The method addresses a key limitation in current structural biology: the difficulty of predicting supramolecular assemblies formed by short peptides, which are often inaccessible to both high-resolution experimental techniques and conventional structure prediction tools. The InRegPep workflow combines efficient CG sampling using the CABS model with symmetry-aware scoring, clustering, and atomistic refinement, enabling the generation of structurally diverse and physically plausible aggregate models. Rather than focusing on a single predicted structure, the approach emphasizes ensemble generation, which is particularly well-suited for capturing the intrinsic polymorphism of amyloid systems. Benchmarking results demonstrate that the method can produce near-native protofilament models for the majority of the 15 tested systems, encompassing a diverse set of experimentally determined amyloid structures, while also highlighting the persistent challenge of accurately ranking candidate conformations.

## Data Availability

The datasets presented in this study can be found in online repositories. The names of the repository/repositories and accession number(s) can be found below: https://zenodo.org/records/20513424 (DOI 10.5281/zenodo.20513423). The InRegPep server is freely available to users as an online tool at https://bioinfo.imdik.pan.pl/inregpep/.
