# Supplementary material for: InRegPep: a web server for modeling of parallel beta-sheet fibrillar aggregates of amyloidogenic peptides
Source: Front Mol Biosci. 2026 Oct 16;13:1908797. doi: 10.3389/fmolb.2026.1908797 (PMC13719315; doi:10.3389/fmolb.2026.1908797)
Supplement: Supplementary file 1 [file Supplementaryfile1.pdf]

Supplementary Material

InRegPep: a web server for modeling of parallel beta-sheet fibrillar aggregates of amyloidogenic peptides

Wojtek Puławski[1], Szymon Niewieczerzał[2], Andrzej Koliński[3], Grzegorz Firlik[1] and Michał Koliński[1,*]

[1] Bioinformatics Laboratory, Mossakowski Medical Research Institute, Polish Academy of Sciences, Warsaw, 02-106, Poland

[2] Biological and Chemical Research Centre, University of Warsaw, Warsaw, 02-089, Poland

[3] Faculty of Chemistry, University of Warsaw, Warsaw, 02-089, Poland

* To whom correspondence should be addressed. Email: mkolinski@imdik.pan.pl

1 Server usage

InRegPep is an online web server for structural modeling of aggregates formed by amyloid-prone peptides that adopt an in-register, parallel β-sheet architecture. Note that this method is not designed to predict whether a given sequence forms amyloids; rather, it is intended to propose aggregate conformations for peptides that are already considered amyloidogenic. The models are generated solely from the input amino acid sequence. The modeling procedure begins with the simultaneous docking of five identical peptide chains using the CABS coarse-grained multichain model [1, 2]. During docking simulations, the interacting peptides are treated as fully flexible, and the CABS model enables highly efficient exploration of the conformational space of emerging peptide complexes [3-5]. Aggregation-specific weak distance restraints are imposed on pairs of corresponding residues in neighbouring chains, promoting an in-register, parallel arrangement. From the large set of predicted models, the top ten-scoring models are selected based on a combination of energetic-based and structural-based criteria. The structural quality and stability of each model are then evaluated during short MD simulations, based on observed patterns of amino acid contacts and the conservation of hydrogen bonds. Accordingly, all models are classified as either "Plausible" or "Unstable." Finally, the "Plausible" structures are used to assemble a larger aggregate, yielding a protofilament model consisting of 15 peptide layers.

Job submission

A job can be submitted using either **Basic** or **Advanced mode**.

Basic mode: The user provides only the amino acid sequence of an amyloidogenic peptide that forms aggregates (sequence length: 10–100 residues; format: uppercase letters only, using one-letter amino acid codes). After submission, the server generates a link to the results page. Optionally, the user may provide an email address, and the link to the result page will be sent by email. The user may also provide a name for the task in the Job name field. By default, modeling results are visible to everyone. To make them private and accessible only to the user who submitted the job, check the appropriate box on the input page.

Advanced mode: The user can modify the following simulation parameters:

a) **Number of docking simulations**: values from 50 to 100 are allowed (default: 50). For short peptides, 50 simulations are usually sufficient. For more complex systems (longer peptides), a higher number of runs is recommended. Increasing this value results in a longer docking trajectory and a larger initial model ensemble for analysis, but also increases the total job runtime on the server.
b) **Monte Carlo Steps**: values from 50 to 100 are accepted (default: 50). For simple systems, 50 steps may be sufficient. Larger systems typically require more MC steps to achieve better sampling of the conformational space.
c) **Disulfide bridges (−S–S−)**: allows the user to impose distance restraints on pairs of cysteine residues that mimic a disulfide bond. Use the format i:j (residue indices separated by semicolon) to define a disulfide bridge between residues. For example, 3:6,11:16 (separated by a comma) denotes two disulfide bridges.
d) **Initial temperature**: sets the simulated annealing temperature range (default: 2.0); values in the range 2.0–3.0 are accepted for the initial temperature.
e) **Final temperature**: sets the simulated annealing temperature range (default: 1.0); values in the range 1.0–1.5 are accepted for the final temperature.
f) **Secondary structure**: allows the user to define a secondary structure assignment for the submitted sequence (C = coil, E = extended). The default value is “C” for the entire peptide sequence. For example, to assign an extended conformation to the first 10 residues and a coil conformation to residues 11 to 15, enter: EEEEEEEEEECCCCC.
g) **pH**: sets the pH value used to define protonation states of peptide amino acids during model reconstruction, molecular dynamics refinement, and scoring.

Running the job

After job submission, a link to the results page is generated. The results are displayed on the Results page once the job is completed. Calculations typically take from a few hours to several days, depending on the length of the submitted sequence and the cluster load.

If an email address was provided, a message containing a link to the results will be sent, along with a notification when the job is completed. The status of the submitted job can be viewed on the Job list page: https://bioinfo.imdik.pan.pl/inregpep/joblist

Viewing the results

After the job is completed, the results are available on the Results page. The simulation parameters and job name are listed at the top of the page.

A plot of CABS-dock energy vs. pcaRMSD is provided for each simulated system, illustrating the relationship between the energy of the forming aggregate [arbitrary units] and the translational symmetry (pcaRMSD) [Å] of interacting peptides.

Structures and additional data for the 10 predicted aggregate models can be displayed using the buttons on the right (Model 1 to Model 10). For each model, the following data and analyses are provided:

a) The 3D structure of the predicted aggregate (displayed in the panel: Models).
b) A bar plot showing RMSD and pcaRMSD values: RMSD describes model conformational stability, while pcaRMSD reflects the translational symmetry of aggregating peptides. Both values are averaged over the final 1 ns of the MD simulation.
c) A bar plot showing normalized β-sheet content and internal contacts between aggregating peptides, calculated over the entire MD simulation.
d) A bar plot showing per-residue β-sheet content, calculated and normalized over the entire MD simulation.
e) A root mean square fluctuation (RMSF) plot showing residue-wise RMSF values calculated over the entire MD simulation. Low RMSF values indicate regions of the aggregating peptide with lower conformational flexibility.
f) Protofilament structures assembled for aggregate models classified as plausible, a 15-layer protofilament models are proposed.

All plots (graphics) and data (including structures) can be downloaded as a single ZIP archive by clicking "Download results." The downloadable ZIP file contains the following:

- **pca_vs_e.png** – A figure illustrating the relationship between pcaRMSD and CABS energy for all models generated during the CABS simulation.
- **summary.txt** – A file containing ranking details for each presented model. It includes pcaRMSD and 10-points ranking, RMSD deviation from the initial structure, the energy gap between each model and the lowest-energy structure in the generated ensemble, and the percentage of β-sheet content. All values (except the energy gap) are averaged over the final 10 snapshots of the 20 ns molecular dynamics trajectory.
- **model1.pdb** to **model10.pdb** – Models of peptide aggregates in PDB format, each containing 5 layers. Models are numbered according to their pcaRMSD rankings.
- **traj.zip** - A compressed PDB-format trajectory of representative aggregate models in Cα-trace representation obtained from all docking trajectories. In particular, it includes the lowest-energy structure, the structure with the highest symmetry (as indicated by the pcaRMSD parameter), and 10 structures identified using k-means clustering for each Monte Carlo run. The total number of unique structures is equal to 12 × the number of Monte Carlo runs.

Every aggregate model is accompanied by:

- A data file with local β-sheet content (per residue) for the modeled peptide aggregate (e.g., **model1.sspr.dat**) and a figure depicting local β-sheet content (e.g., **model1.sspr.png**).
- A data file with root mean square fluctuations (RMSF) calculated during 20 ns of molecular dynamics simulation. It contains data for each peptide layer separately, as well as values

averaged over all 5 layers (e.g., **model1.rmsf.dat**). There is also a figure depicting RMSF for each peptide residue (e.g., **model1.rmsf.png**), which shows how much each residue in each layer fluctuates around its average position over time.

- A contact map figure (e.g., **model1.cmap.png**) — a 2D matrix showing which Cα atoms within a single layer are in close physical proximity in the 3D structure.
- A short protofilament model consisting of 15 interacting peptides (e.g., **protofilament1.pdb**), provided in PDB format. This model is generated only for aggregate models classified as "plausible" and is constructed based on structural regularities observed in predicted models. The peptide from the aggregate central layer is used as the building block for protofilament assembly. Successive layers are generated along the protofilament's long (Z) axis by replicating this peptide and positioning each copy using a transformation matrix derived from neighbouring layers. The resulting protofilament model is then refined by geometry optimization using implicit-solvent molecular dynamics.

Additional information:

Data Availability

- The InRegPep method is freely accessible through the web server at: https://bioinfo.imdik.pan.pl/inregpep/
- The InRegPep server source code is available at: https://zenodo.org/records/20513424
- The benchmark dataset, including the protofilament sequences used for test predictions, is available at: https://bioinfo.imdik.pan.pl/inregpep/benchmark

Examples

- Two examples demonstrating how to predict protofilament structures using InRegPep are available at: https://bioinfo.imdik.pan.pl/inregpep/examples. At the bottom of the page, a link directs users to a preloaded submission page containing example data ready for calculation.

Tutorial

- A tutorial describing the use of the server is available at: https://bioinfo.imdik.pan.pl/inregpep/tutorial
- Additional guidance on using the server and interpreting the results can be found on the Questions & Answers page at: https://bioinfo.imdik.pan.pl/inregpep/qanda

Authors

For comments, questions, or feedback, please contact:

- Wojciech Puławski, PhD — wpulawski@imdik.pan.pl
- Szymon Niewieczerzał, PhD — sz.niewieczerzal@cent.uw.edu.pl
- Michał Koliński, PhD — mkolinski@imdik.pan.pl

Funding

This work was supported by the National Science Center of Poland (NCN), grant no. 2021/43/B/NZ2/02082.

License

This project is licensed under the MIT License.

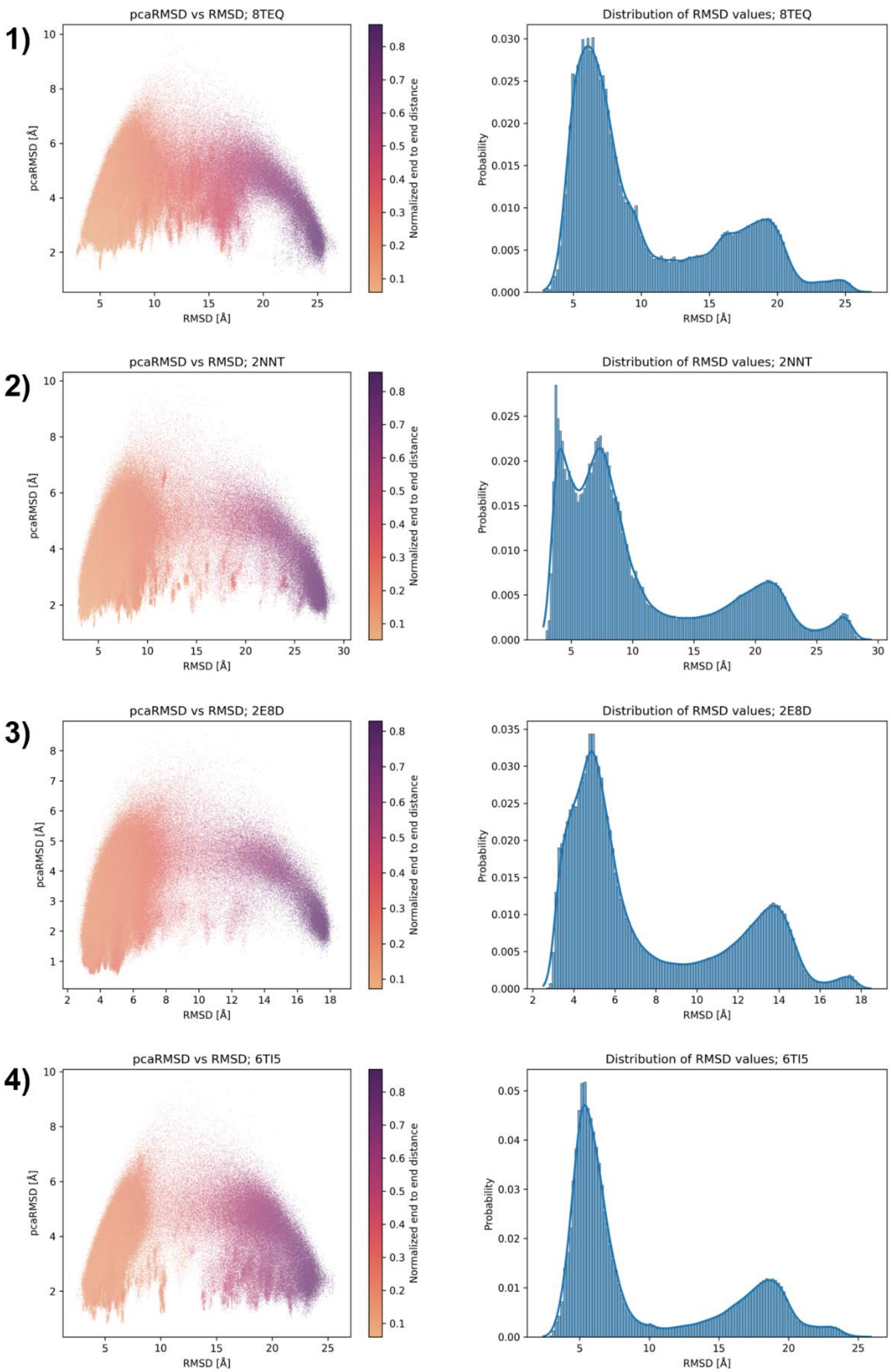
1)
pcaRMSD vs RMSD; 8TEQ
Distribution of RMSD values; 8TEQ
2)
pcaRMSD vs RMSD; 2NNT
Distribution of RMSD values; 2NNT
3)
pcaRMSD vs RMSD; 2E8D
Distribution of RMSD values; 2E8D
4)
pcaRMSD vs RMSD; 6TI5
Distribution of RMSD values; 6TI5
pcaRMSD [Å]
RMSD [Å]
Normalized end to end distance
Probability

5)

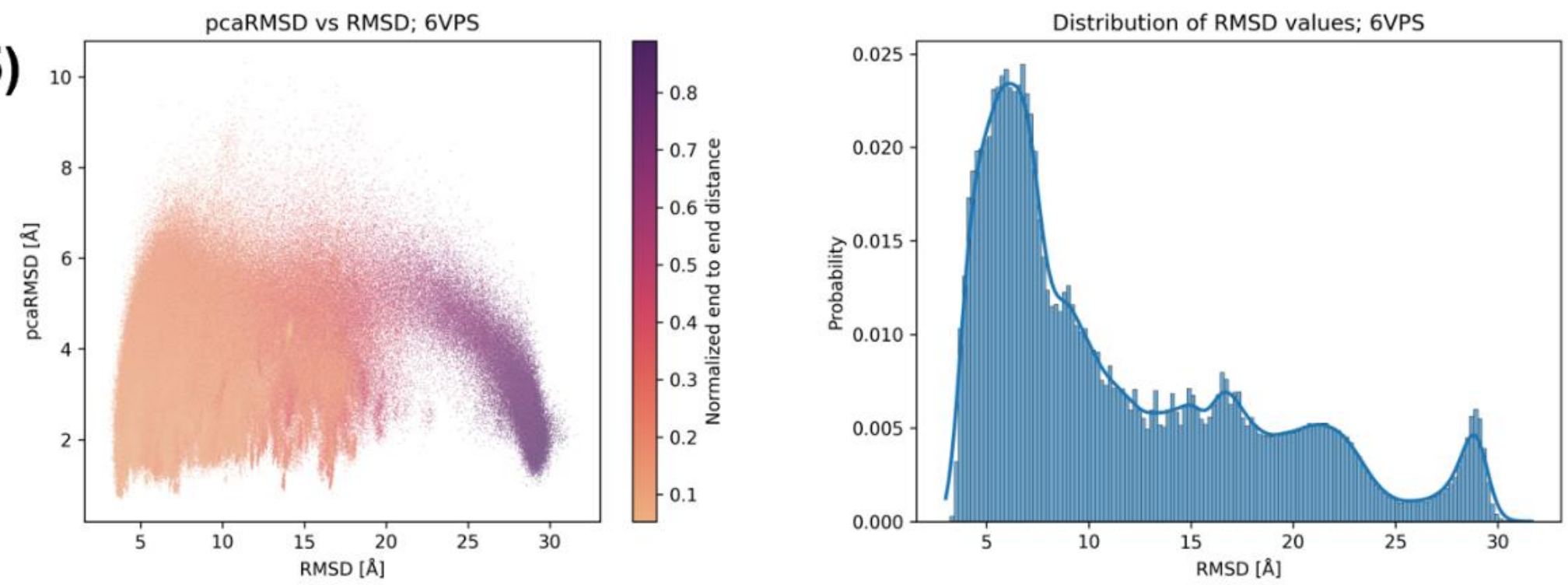
pcaRMSD vs RMSD; 6VPS
pcaRMSD [Å]
RMSD [Å]
Normalized end to end distance
Distribution of RMSD values; 6VPS
Probability
RMSD [Å]

6)

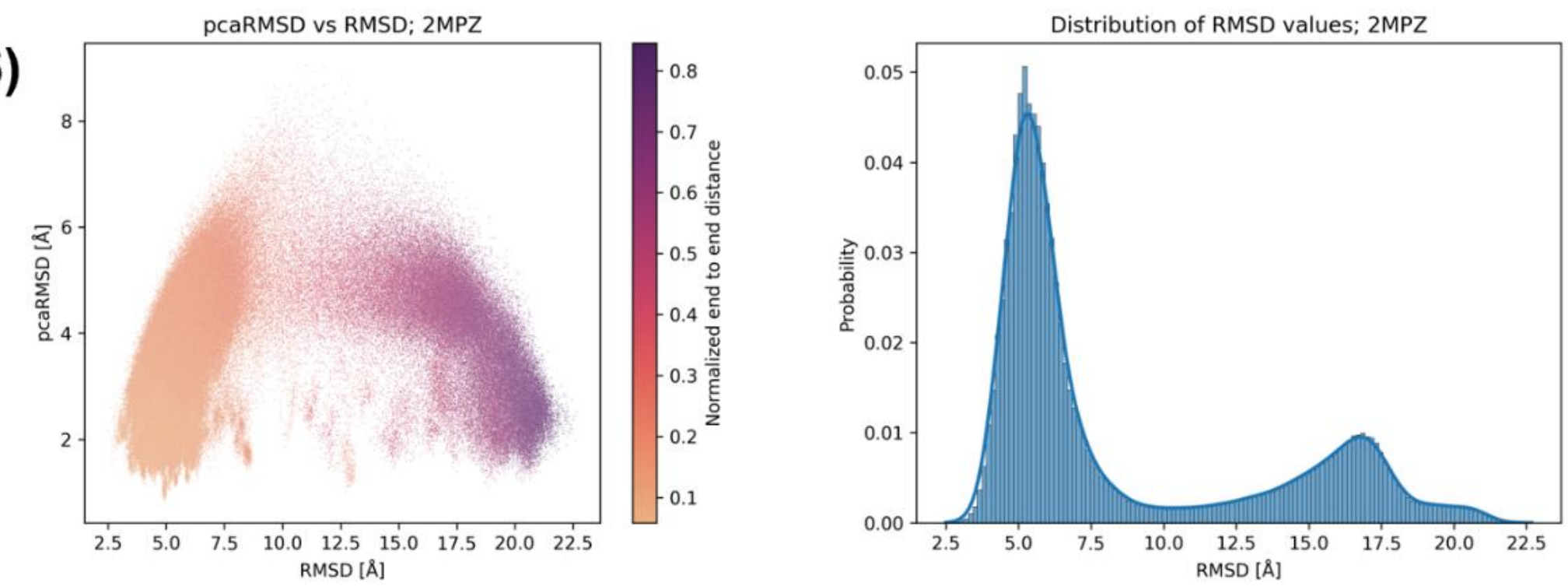
pcaRMSD vs RMSD; 2MPZ
pcaRMSD [Å]
RMSD [Å]
Normalized end to end distance
Distribution of RMSD values; 2MPZ
Probability
RMSD [Å]

7)

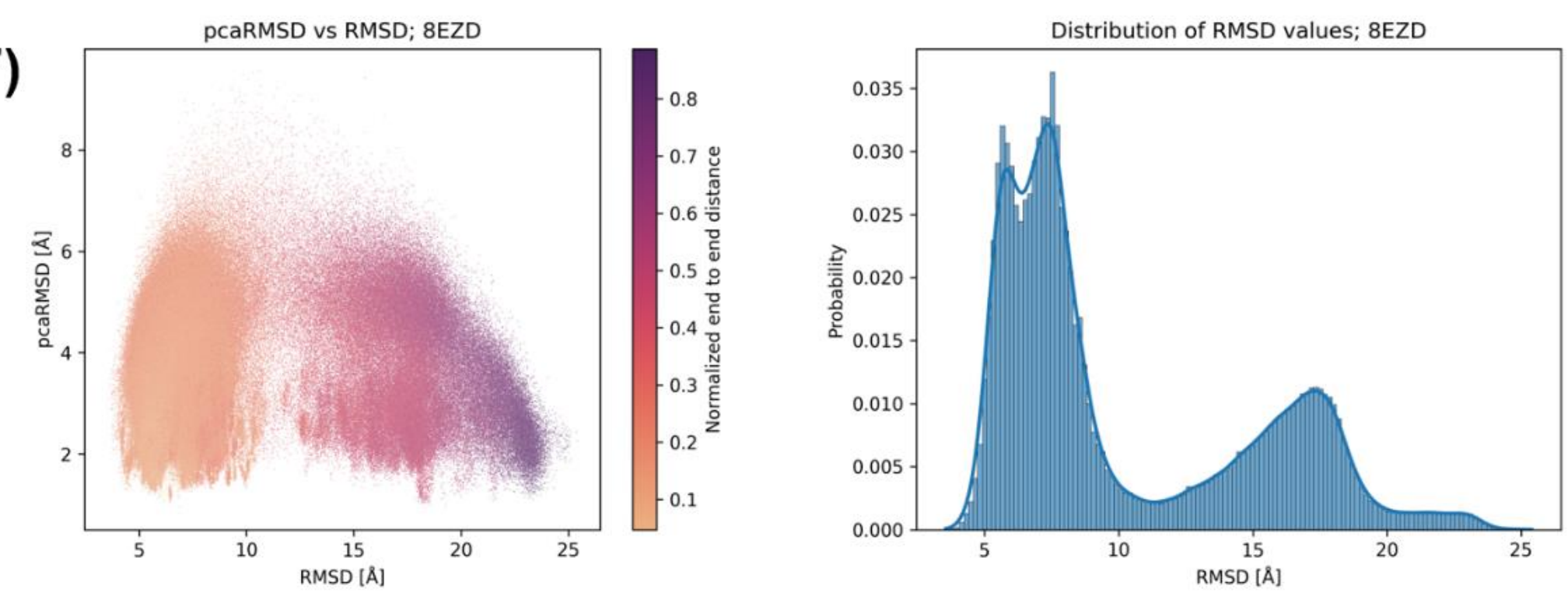
pcaRMSD vs RMSD; 8EZD
pcaRMSD [Å]
RMSD [Å]
Normalized end to end distance
Distribution of RMSD values; 8EZD
Probability
RMSD [Å]

8)

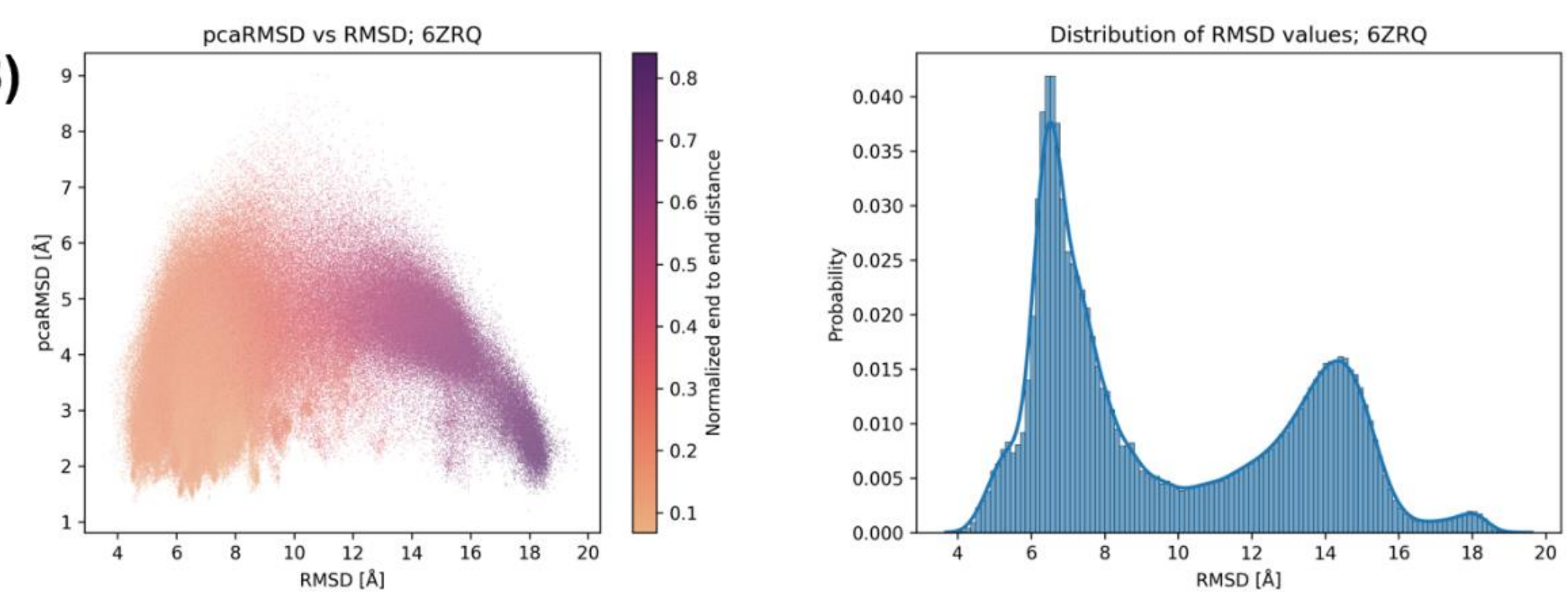
pcaRMSD vs RMSD; 6ZRQ
pcaRMSD [Å]
RMSD [Å]
Normalized end to end distance
Distribution of RMSD values; 6ZRQ
Probability
RMSD [Å]

9)

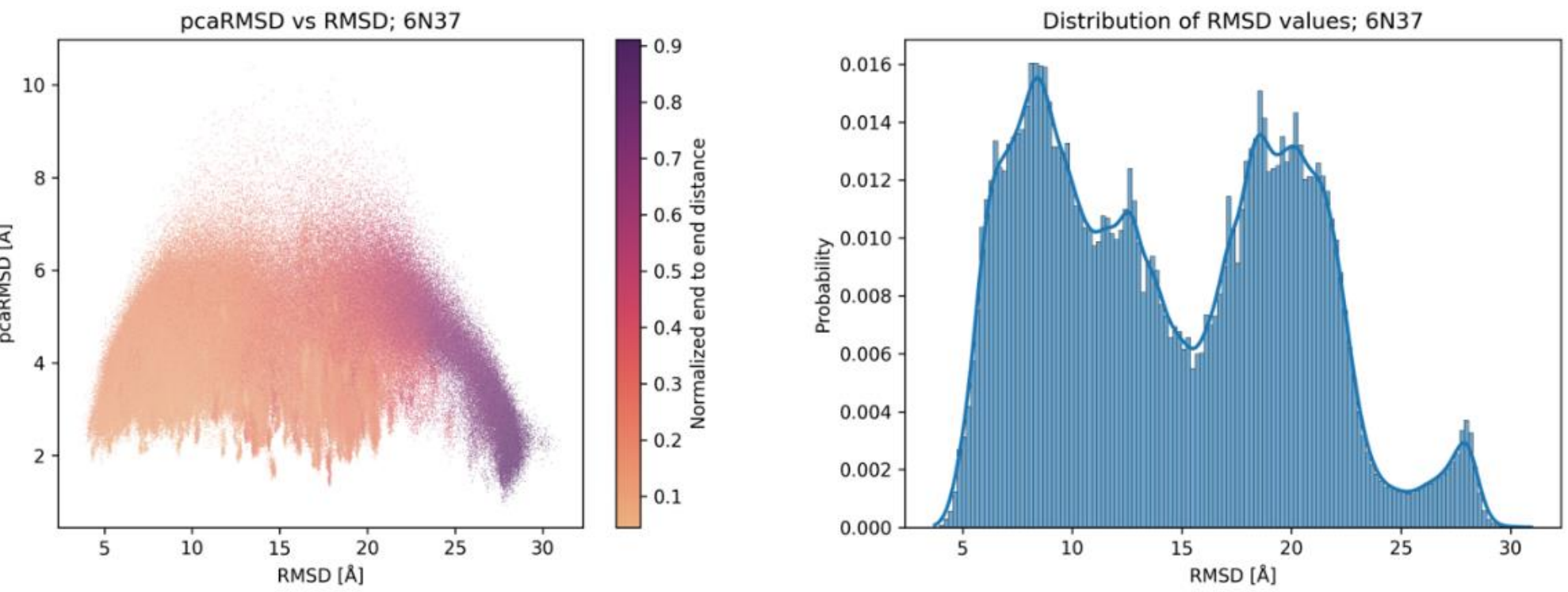

pcaRMSD vs RMSD; 6N37
pcaRMSD [Å]
RMSD [Å]
Normalized end to end distance
Distribution of RMSD values; 6N37
Probability
RMSD [Å]

10)

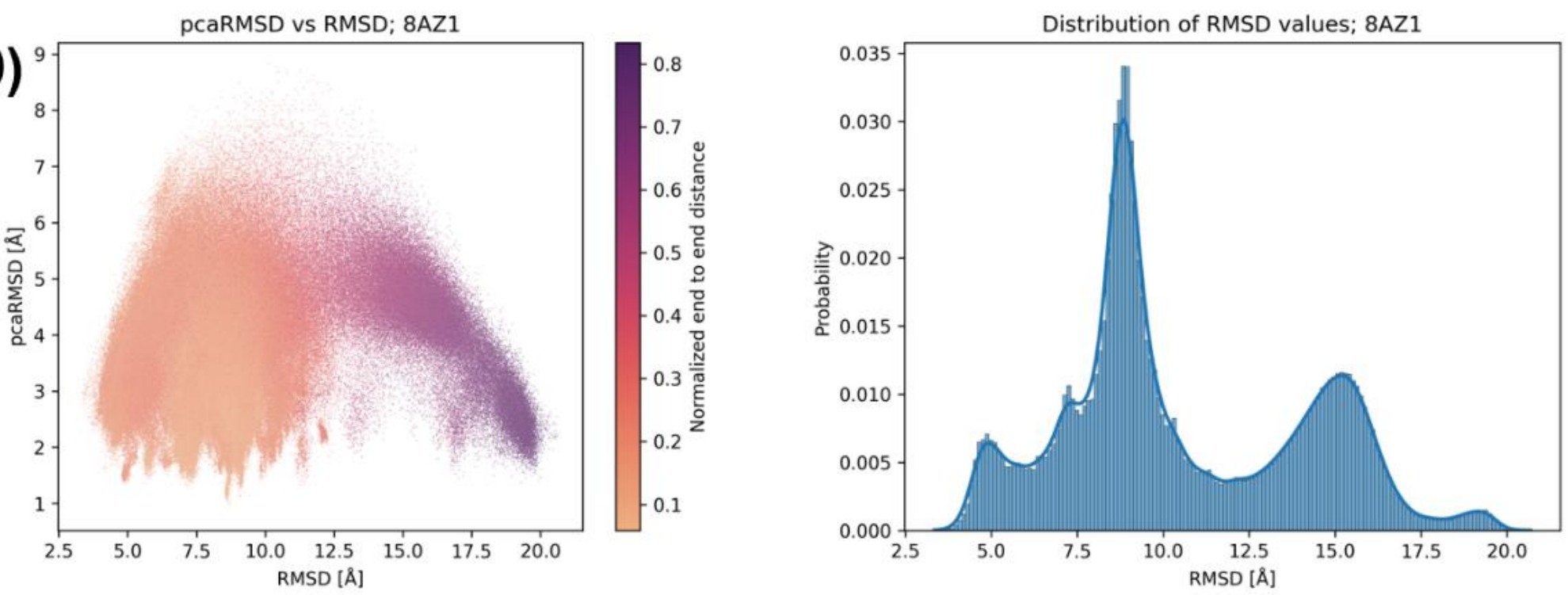

pcaRMSD vs RMSD; 8AZ1
pcaRMSD [Å]
RMSD [Å]
Normalized end to end distance
Distribution of RMSD values; 8AZ1
Probability
RMSD [Å]

11)

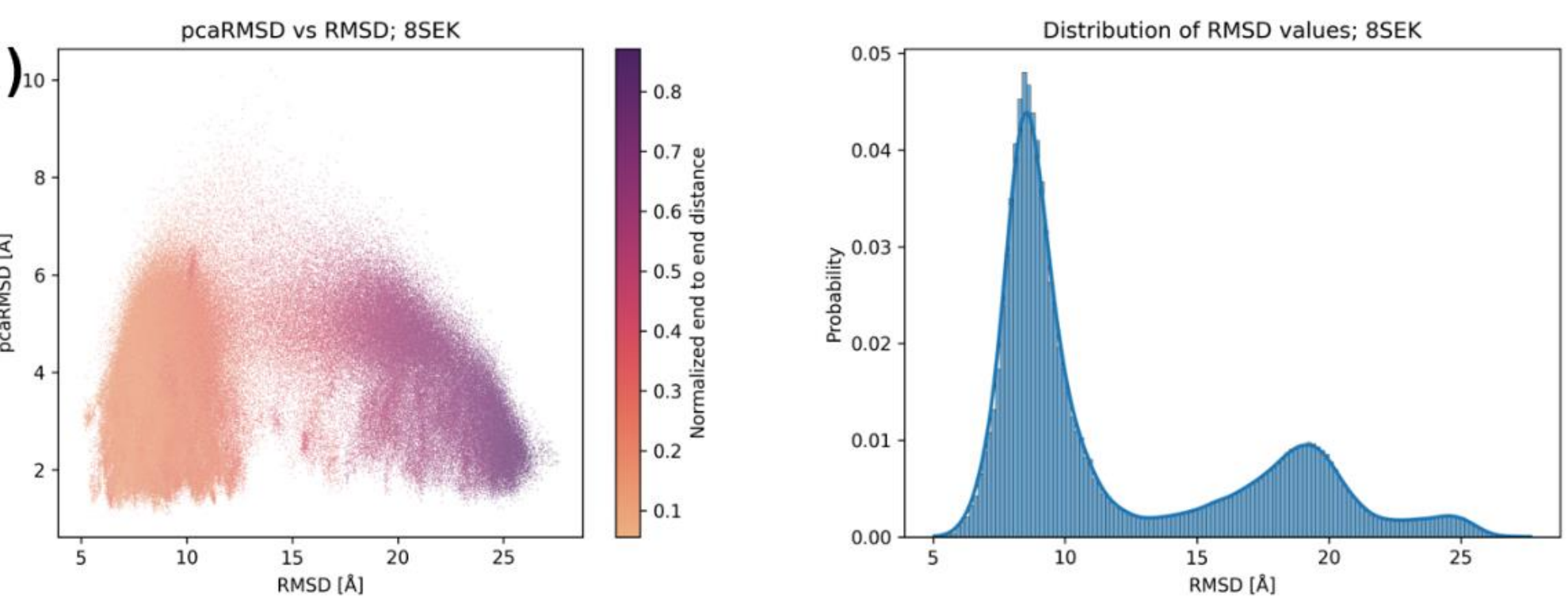

pcaRMSD vs RMSD; 8SEK
pcaRMSD [Å]
RMSD [Å]
Normalized end to end distance
Distribution of RMSD values; 8SEK
Probability
RMSD [Å]

12)

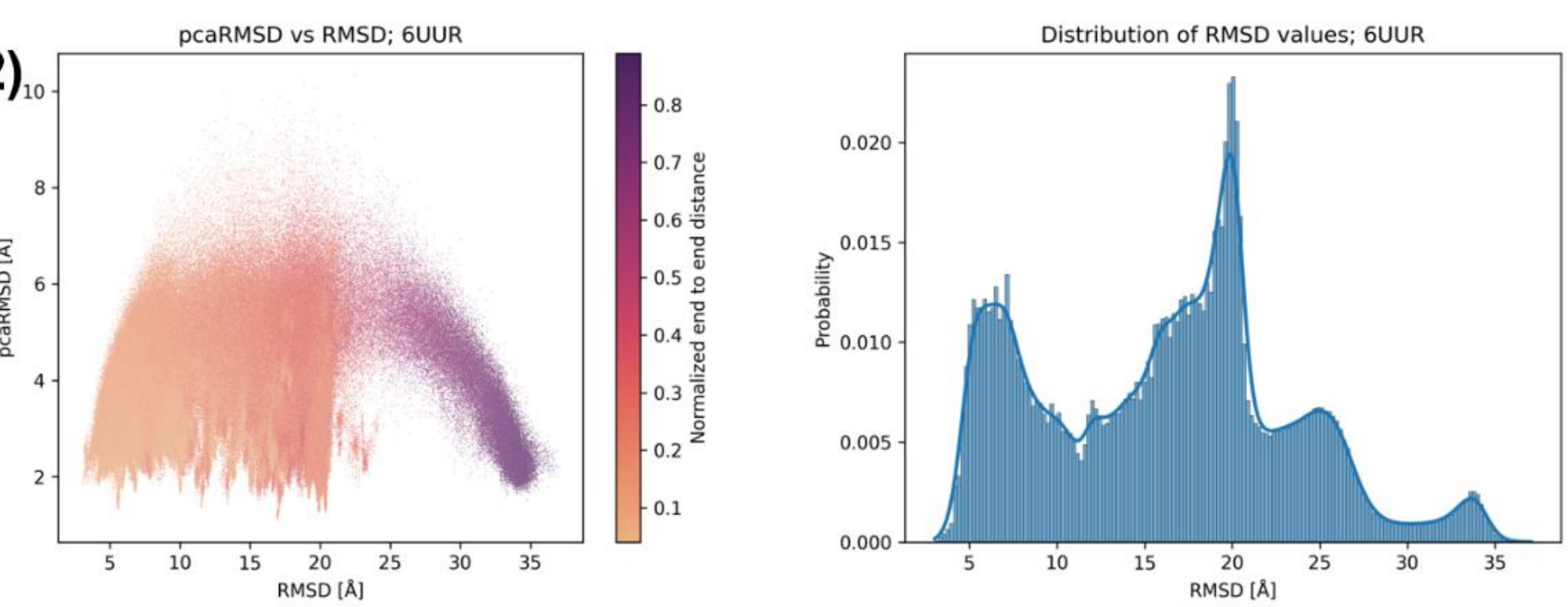

pcaRMSD vs RMSD; 6UUR
pcaRMSD [Å]
RMSD [Å]
Normalized end to end distance
Distribution of RMSD values; 6UUR
Probability
RMSD [Å]

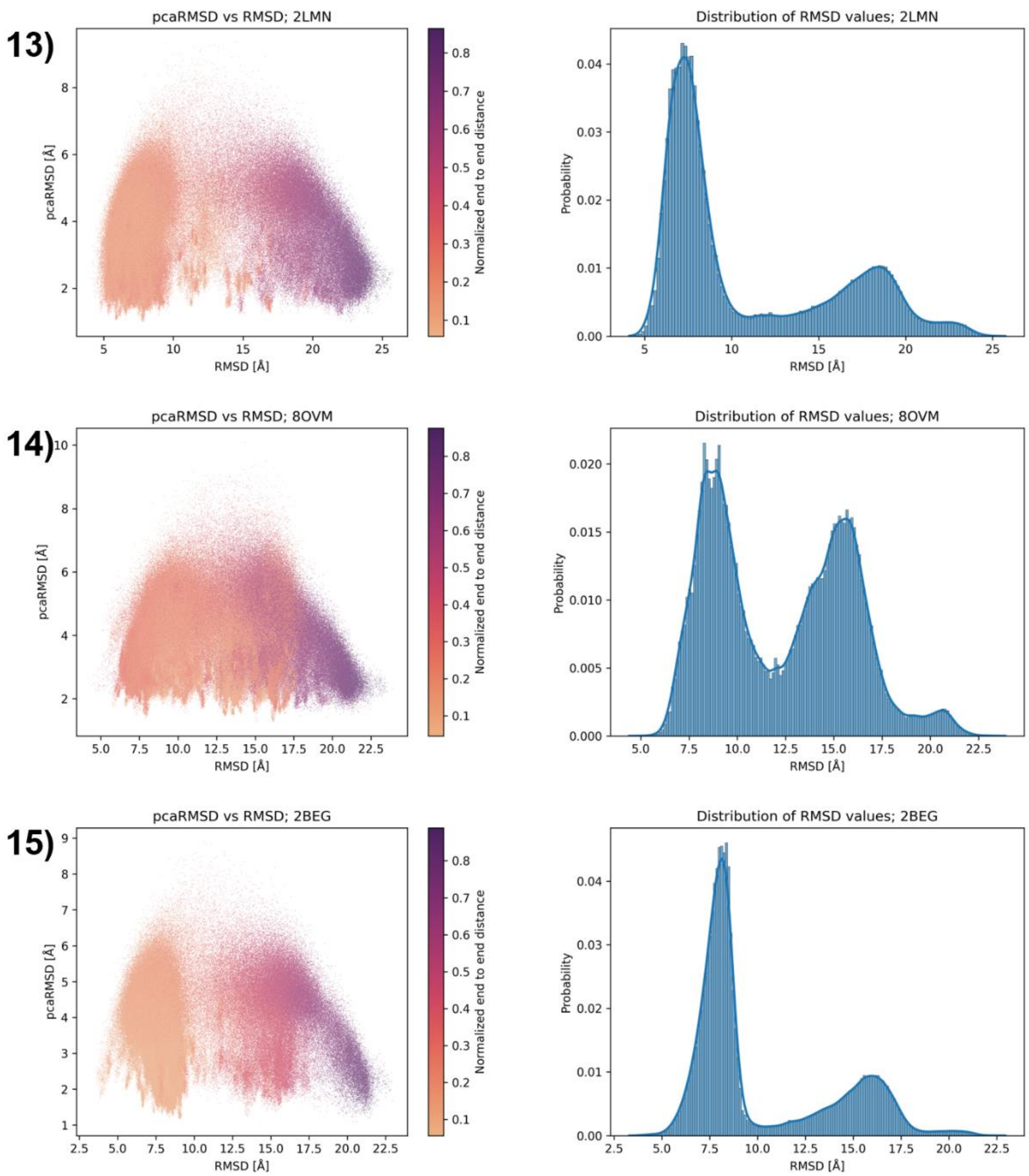

Figure S1. Analysis of the quality of predicted models based on RMSD values calculated for the benchmark dataset. The left panel shows the correlation between the pcaRMSD and RMSD parameter values for all generated aggregate models during the benchmark test prediction. The pcaRMSD depicts the translational symmetry of peptide layers in each aggregate and is one of the main criteria for best model selection. The right panel shows the distribution of RMSD values calculated for all generated models obtained for each system. The above data indicate that, in most cases, the sampling procedure is sufficient to generate models with low RMSD values. The low number of high-quality models in proportion to the entire trajectory, together with the insufficient correlation between the scoring function and model quality, makes it hard to identify a single accurate model for each prediction. For example, see model number 7, despite the presence of high-quality models in the trajectory (see Table 1; "Best from all" shows 3.55 Å), the scoring procedure was not able to identify a single best model — the best-scored model showed an RMSD value of 18.31 Å. At the same time, we were able to select 10 top-scored models, among which a model below 5 Å was included. A similar

situation was present for model 9 (see Table 1). The scoring procedure was able to identify a single high-quality model for only four systems (where, by high-quality model, we define a model with RMSD values below 5 Å). Because of this, we report sets of the 10 top-scored models, from which we report the model with the lowest RMSD value.

Table S1. Success rates for the benchmark data. Random selection reports the fraction of high quality models (e.g., models with RMSD < 5.0 Å) among all generated models, column 3. In contrast, Top-k reports the number of high quality models among sets of k highest-scoring models, with k = 1, 10, and 100, (columns 4 to 6). The success rate calculated for entire benchmark is 0.33, for Top-1 (best-scored model), 0.67 for Top-10, and 0.80 for Top-100. Here, the success rate is defined as the probability that at least one good model is found among the sets of highest-ranked models (k = 1, 10 or 100).

**N.o.**	**Model**	**Random selection**	**Top-1**	**Top-10**	**Top-100**
1	8TEQ	0.089	1	6	39
2	2NNT	0.191	1	8	74
3	2E8D	0.359	0	9	88
4	6TI5	0.171	0	4	40
5	6VPS	0.118	1	5	33
7	2MPZ	0.200	1	4	48
8	8EZD	0.021	0	1	3
9	6ZRQ	0.015	1	1	8
10	6N37	0.006	0	1	6
11	8AZ1	0.038	0	1	7
12	8SEK	0.000	0	0	0
13	6UUR	0.027	0	0	10
14	2LMN	0.001	0	0	2
15	8OVM	0.000	0	0	0
16	2BEG	0.002	0	0	0

Table S2. Structural descriptors for the benchmark data including β-sheet content and protofilament twist angle. The reference structures generally exhibit higher secondary-structure ratios than our models. However, it should be noted that our models were not subjected to any rigorous optimization procedure. The best models identified from the trajectories (with the lowest RMSD value) more frequently deviate from the reference structure than the best-scoring models. This may be due to the fact that the scoring function preferentially selects models with highest translational symmetry but not always with the optimal geometry of interacting peptides. The observed relationship with RMSD is relatively weak, suggesting that these descriptors alone are only modestly indicative of overall structural quality. The β-sheet ratio was defined as the percentage of Cα atoms involved in β-sheet structures, as determined by the STRIDE algorithm. The twist angle was determined from the rotational component of the transformation matrix, obtained by transforming the upper *n*-1 layers onto the lower *n*-1 layers, where *n* denotes number of layers in the given structure.

Model	β-sheet content				Twist angle [º]				RMSD [Å]		
	Best from all	Best from top 10	Best scored	Reference	Best from all	Best from top 10	Best scored	Reference	Best from all	Best from top 10	Best scored
8teq	0.26	0.23	0.26	0.59	2.52	3.91	3.56	3.07	2.81	2.84	5
2nnt	0.42	0.34	0.43	0.69	4.01	3.96	1.48	1.04	2.74	2.97	4.59
2e8d	0.32	0.43	0.37	0.52	4.74	4.11	4.74	4.54	2.52	3.36	5.12
6ti5	0.51	0.48	0.41	0.71	4.07	1.14	2.09	1.2	2.4	3.64	5.17
6vps	0.32	0.56	0.6	0.62	2.28	0.93	1.2	1.53	3.01	3.71	3.77
2mpz	0.36	0.38	0.38	0.58	1.8	2.38	2.42	2.21	2.5	4.09	4.96
8ezd	0.41	0.33	0.43	0.52	2.6	4.34	0.75	1.73	3.55	4.44	18.31
6zrq	0.19	0.44	0.43	0.52	1.14	3.37	2.82	1.99	3.67	4.62	4.62
6n37	0.16	0.26	0.26	0.52	3.17	1.46	2.85	2.8	3.73	4.84	17.9
8az1	0.23	0.45	0.41	0.6	4.57	3.93	2.75	2.12	3.34	4.91	8.67
8sek	0.31	0.38	0.62	0.5	6.29	1.43	2.4	0.46	5.04	5.41	9.22
6uur	0.34	0.22	0.36	0.62	2.25	3.26	2.64	1.38	3.03	5.47	17.01
2lmn	0.43	0.47	0.6	0.45	3.33	2.32	3.61	2.5	4.12	5.89	7.75
8ovm	0.35	0.25	0.42	0.58	2.14	1.21	1.09	2.26	4.4	7.3	16.15
2beg	0.48	0.48	0.32	0.77	1.4	2.42	1.58	1.78	3.29	7.38	9

References

1. Kurcinski. M. and A. Kolinski. *Hierarchical modeling of protein interactions.* J Mol Model. 2007. **13**(6-7): p. 691-8.
2. Kurcinski. M.. A. Badaczewska-Dawid. M. Kolinski. A. Kolinski. and S. Kmiecik. *Flexible docking of peptides to proteins using CABS-dock.* Protein Sci. 2020. **29**(1): p. 211-222.
3. Puławski. W.. A. Koliński. and M. Koliński. *Multiscale modeling of protofilament structures: A case study on insulin amyloid aggregates.* International Journal of Biological Macromolecules. 2025. **285**: p. 138382.
4. Koliński. M.. R. Dec. and W. Dzwolak. *Multiscale modeling of amyloid fibrils formed by aggregating peptides derived from the amyloidogenic fragment of the a-chain of insulin.* International journal of molecular sciences. 2021. **22**(22): p. 12325.
5. Puławski. W.. A. Koliński. and M. Koliński. *Integrative modeling of diverse protein-peptide systems using CABS-dock.* PLOS Computational Biology. 2023. **19**(7): p. e1011275.
